# 

**Published:** 1882-10

**Authors:** 


CONTENTS:

ART. I.—Diagnosis and Treatment of Benign Tumors. By G. H. Boyland, M. D....241

ART. II.—Histology of Nerves. A Contribution of the Latest Researches. By L. D Beane, M. D....254

ART. III.—Proceedings of American Dental Association (Continued)....260

ART. IV.—Review of the Progress in Histology. By Dr. E. S. Davenport....268

ART. V.—Asphyxiated by a Tooth. By N. Miller. D. D. S....278

ART. VI.—The Cause of Death....279

EDITORIAL, ETC.

The Auspicious Opening of the first Regular Session of the Dental Department of the University of Maryland....283

MONTHLY SUMMARY.

The Use of Tobacco by Boys....283
Scurvy and Fresh Meat....284
Ether....285
Microscopic Structure of Malleable Metals....286
No Salivation from Amalgam Fillings....287
Cement for Rubber....287
Crural Neuralgia among Dentists....287

